# Maternal and Perinatal Outcomes in Pregnant Women with Cancer: A Single-Center Retrospective Cohort Study

**DOI:** 10.3390/diagnostics15081012

**Published:** 2025-04-16

**Authors:** Bruna Elias Parreira Lopes Ferraz, Roney César Signorini Filho, Lucas Ribeiro Borges Carvalho, Michelle Samora Almeida, Tatiana Carvalho de Souza Bonetti, Edward Araujo Júnior, Antonio Braga, Sue Yazaki Sun, Roberta Granese

**Affiliations:** 1 Department of Obstetrics, Paulista School of Medicine, Federal University of São Paulo (EPM-UNIFESP), Sao Paulo 04023-062, Brazil; brunaeliasparreira@gmail.com (B.E.P.L.F.); dr.roneycesar@gmail.com (R.C.S.F.); lucasrbc@hotmail.com (L.R.B.C.); araujojred@terra.com.br (E.A.J.); sueysun@gmail.com (S.Y.S.); 2 Discipline of Oncology, Paulista School of Medicine, Federal University of São Paulo (EPM-UNIFESP), Sao Paulo 04023-062, Brazil; msoncologia@hotmail.com; 3 Department of Gynecology, Paulista School of Medicine, Federal University of São Paulo (EPM-UNIFESP), Sao Paulo 04023-062, Brazil; tbonetti@unifesp.br; 4 Department of Obstetrics and Gynecology, Federal University of Rio de Janeiro (UFRJ), Rio de Janeiro 22240-001, Brazil; bragamed@yahoo.com.br; 5 Department of Biomedical and Dental Sciences and Morphofunctional Imaging, “G. Martino” University Hospital, 98100 Messina, Italy

**Keywords:** pregnancy, cancer, chemotherapy, survival, perinatal outcomes

## Abstract

**Objective:** The aim of our study was to evaluate maternal and perinatal outcomes in pregnant women diagnosed with cancer and treated at a single referral center in Brazil. **Methods:** This retrospective cohort study analyzed medical records from January 2008 to December 2020. Demographic, clinical, obstetric, and tumor-related variables were assessed. Patients were divided into two groups: Group 1 (*n* = 28) included women diagnosed with cancer during pregnancy or up to one year postpartum, while Group 2 (*n* = 11) comprised those who became pregnant during cancer investigation or treatment. **Results:** The most prevalent cancers were breast (G1 = 11, G2 = 3), cervical (G1 = 10, G2 = 3), and hematologic (G1 = 2, G2 = 4). Treatment modalities included surgery (*n* = 11), chemotherapy (*n* = 21), and inadvertent radiotherapy in one case. Most newborns (*n* = 25) were delivered at term, with one miscarriage, one fetal death, and one neonatal death reported. Thirty-two newborns were appropriate for gestational age, and thirty-seven were discharged with their mothers. Preterm delivery was indicated for obstetric reasons in 61.5% of cases. Overall survival by cancer type was 54% for breast, 70% for cervical, and 100% for hematologic cancers. The total survival rate was 70.9%. **Conclusions:** Cervical cancer was the second most common type in this cohort. Most deliveries occurred at term, and newborns were adequate for gestational age. Despite cancer treatment during pregnancy, most neonates were discharged alongside their mothers.

## 1. Introduction

In recent decades, a growing incidence of cancer has been observed worldwide, including among women of reproductive age. According to estimates from the World Health Organization (WHO), over nine million new cases of cancer in women occurred globally in 2020 [1]. In Brazil, the National Cancer Institute (INCA) estimates 316,280 new cases of cancer in women for the same year [2]. This trend has led to an increase in cases where cancer coexists with pregnancy, a condition that presents complex clinical and ethical challenges [3].

Cancer in pregnancy is commonly defined as any malignancy diagnosed during pregnancy or within one year after delivery [4]. Although relatively rare, with an estimated incidence of 1 case in 1000 pregnancies, several population-based studies have reported an upward trend in its occurrence [3,5,6]. For instance, a Danish study reported an increase from 572 cases in the period 1977–1986 to 1052 in the period 1997–2006, suggesting a proportional rise from 5.4% to 8.3% among pregnancy-related cancers [7].

Delayed childbearing is considered one of the main contributing factors to this rise [3,8]. Eibye et al. [7] showed that 63.8% of cancers in pregnancy occurred in women over the age of 30 years. However, other factors such as improved diagnostic techniques, greater awareness among healthcare professionals, and increased access to prenatal and postnatal care also appear to play a significant role [9].

Despite the growing global interest in this topic, there is limited research addressing maternal and perinatal outcomes in pregnancy-associated cancer within low- and middle-income countries. The Brazilian context, marked by regional disparities in healthcare access and resources, presents a unique opportunity to examine these outcomes in a real-world setting.

Although international studies have described maternal and neonatal outcomes in women diagnosed with cancer during pregnancy, data from Latin America remain scarce. In Brazil, few published studies have addressed this topic, most with small sample sizes or focused on specific tumor types.

The objective of this study was to evaluate maternal and perinatal outcomes in pregnant women diagnosed with cancer and treated at a single referral center in Brazil. By describing the clinical profiles, treatment strategies, and pregnancy outcomes in this population, the present study aims to contribute to the scarce literature available from developing countries and to inform clinical management in similar healthcare contexts.

## 2. Methods

### 2.1. Study Design and Participants

This retrospective cohort study was conducted at the Pregnancy Neoplasms Sector of the Federal University of São Paulo through the review of medical records covering the period from January 2008 to December 2020. This study was approved by the institutional ethics committee (CAAE: 12770918.0.0000.5505). All living patients included in the analysis were contacted by telephone and invited to participate. Those who agreed signed an informed consent form authorizing the use of their clinical data for research purposes. For deceased patients, data were included based on ethics committee approval and institutional policies for retrospective chart review. All data were anonymized during analysis to preserve confidentiality, and all researchers involved signed a confidentiality agreement to ensure data protection.

Eligible participants were pregnant women who either received a cancer diagnosis during pregnancy or within one year postpartum or who became pregnant while undergoing cancer investigation or active oncologic treatment. Based on these criteria, the sample was divided into two groups: Group 1 included women diagnosed with cancer during pregnancy or within 12 months after delivery, and Group 2 included women who were already undergoing cancer evaluation or treatment at the time of conception.

Women were excluded from the study if the cancer diagnosis had occurred prior to pregnancy and no treatment had been administered during gestation. Additionally, cases with incomplete or missing medical records, which compromised the reliability of data collection, were also excluded.

A total of 67 medical records were initially identified. After applying the exclusion criteria, 28 cases were excluded—23 due to cancer diagnosis preceding pregnancy without treatment during gestation and 5 due to incomplete medical records. Consequently, 39 women were included in the final analysis. Of these, seven cases of cervical cancer had already been published in a previous focused study [10].

### 2.2. Studied Variables

Demographic variables included maternal age at the first prenatal visit; self-reported ethnicity (categorized as white, black, mixed, Asian, or Indigenous, according to the Brazilian Institute of Geography and Statistics—IBGE) [11]; marital status (single or married, with stable unions classified as married); educational level (ranging from illiteracy to completed higher education, based on IBGE classifications); and place of residence (within or outside the state of São Paulo).

Clinical variables comprised family and personal history of cancer, pre-pregnancy body mass index (BMI) adjusted for gestational age (calculated using the Ministry of Health’s prenatal care guidelines) [12], and lifestyle factors including smoking, alcohol consumption, and use of other substances. The contraceptive method used prior to pregnancy was also recorded.

Obstetric variables included the number of previous pregnancies and deliveries, gestational age at the first prenatal visit (calculated from the last menstrual period and confirmed by first-trimester ultrasound using crown–rump length), mode of delivery (vaginal or cesarean), pregnancy and delivery-related complications, gestational age at birth, birth weight, and presence of fetal growth disorders (classified according to Hadlock’s curve) [13].

Tumor-related variables included cancer type (cervical, breast, ovarian, hematologic, gastrointestinal, or bladder), stage (based on clinical, surgical, and/or radiological findings), and treatment modality (surgery, chemotherapy, radiotherapy, or immunotherapy). For patients who received chemotherapy, data were collected on the specific drugs used, dosage, number of cycles, and gestational age at the beginning and end of treatment. In the case of immunotherapy, the timing and duration of exposure during pregnancy were recorded. Additionally, the time interval between cancer and pregnancy diagnoses, as well as information on maternal overall survival and disease-free survival, were included when available.

Cancer staging was classified according to the TNM system for solid tumors and based on standard hematologic classifications for leukemias and lymphomas, as documented in the medical records. In cases where explicit staging criteria were not recorded, staging information was inferred from available clinical, laboratory, and imaging data by consensus between the oncologists involved in this study.

### 2.3. Statistical Analysis

Descriptive statistics were used to summarize the study variables. Categorical variables were presented as absolute and relative frequencies, while continuous variables were expressed as means, medians, and standard deviations, depending on data distribution.

Comparisons between groups were conducted using Fisher’s exact test for categorical variables. For continuous variables, the Mann–Whitney U test was applied, as most variables did not follow a normal distribution.

Survival analysis was performed to evaluate time-to-event outcomes, considering both overall survival and event-free survival. The Kaplan–Meier method was used to estimate survival functions, and comparisons between groups were made using the log-rank test (Mantel–Cox). When differences were observed among cancer types, post hoc comparisons were conducted using the Bonferroni correction.

All statistical analyses were performed using SPSS version 20.0 (Chicago, IL, USA) and STATA version 12 (College Station, TX, USA). A two-tailed *p*-value < 0.05 was considered statistically significant.

## 3. Results

A total of 67 medical records were evaluated, of which 28 were excluded—23 due to cancer diagnosis prior to pregnancy without oncologic treatment during gestation and 5 due to incomplete data. The final sample comprised 39 pregnant women with cancer, divided into two groups: Group 1 (*n* = 28), consisting of women diagnosed with cancer during pregnancy or up to one year postpartum, and Group 2 (*n* = 11), including those who became pregnant during cancer investigation or treatment.

### 3.1. Sociodemographic Characteristics

The mean maternal age was 32.2 years in Group 1 and 30.4 years in Group 2. The majority of participants identified as white (53.9%) and were married or in a stable union (76.9%). Most had completed high school (53.9%) and resided in the state of São Paulo (92.3%). A statistically significant difference in education level was observed between the groups (*p* = 0.013), with Group 1 showing a higher proportion of incomplete primary education (Table 1).

### 3.2. Cancer Types and Staging

Breast cancer (35.9%) and cervical cancer (33.4%) were the most frequent diagnoses, followed by hematologic malignancies (15.4%). Gynecologic cancers predominated in both groups, with a higher proportion observed in Group 1 (82.2%). In contrast, hematologic cancers were more frequent in Group 2 (36.3%) (Table 2).

Advanced-stage disease (stages III and IV) was more common among breast cancer patients in Group 1. In cervical cancer, all cases in Group 2 were diagnosed at stage I, whereas Group 1 included more advanced stages (II and III). Ovarian, gastrointestinal, and bladder cancers were diagnosed at early stages. Among hematologic malignancies, chronic myeloid leukemia was the most frequent, and staging data were not applicable or unavailable in several cases (Table 3).

### 3.3. Clinical and Obstetric History

A significant proportion of patients were classified as overweight (17.9%) or obese (35.9%), particularly those with breast and cervical cancer. Family history of cancer was more frequently reported among women with breast, ovarian, and gastrointestinal tumors (21.4%, 33.3%, and 50%, respectively). Most women denied smoking or drug use during pregnancy (64.1%) (Table S1).

Cervical cancer patients tended to have a higher number of previous pregnancies and deliveries, while breast cancer patients had lower parity. The average age at first sexual intercourse was 16.3 years, with lower means observed in cervical and hematologic cancer cases. One patient reported first intercourse at the age of 9 and was diagnosed with stage IIIC1 cervical cancer at the age of 17 years old.

Contraceptive failure was reported in 80% of Group 2 patients, predominantly among users of combined oral contraceptives (62.5%) and male condoms (37.5%).

### 3.4. Cancer Diagnosis and Treatment During Pregnancy

In Group 1, the mean gestational age at cancer diagnosis was 15.9 weeks. Most diagnoses occurred in the first or second trimester, except for gastrointestinal and cervical cancers, which were more often diagnosed later in pregnancy or postpartum.

Treatment modalities included chemotherapy (38.5%), surgery (12.8%), and combined approaches (15.4%). One patient received unintentional radiotherapy in early pregnancy. Patient G1-13 was recommended neoadjuvant chemotherapy but did not receive it. Chemotherapy protocols varied by cancer type and gestational age.

### 3.5. Maternal and Perinatal Outcomes

Most births occurred at term (65.8%), and 86.5% of newborns were classified as appropriate for gestational age (AGA). Preterm deliveries were mostly indicated for obstetric (61.5%) or oncologic (38.5%) reasons. Among the patients with late preterm labor for obstetric indications, patient G2-7 had her third cycle of chemotherapy interrupted because of suspected fetal growth restriction (FGR) and her labor was induced at 36 weeks. Patient G1-9 had severe pre-eclampsia with FGR. Among the small-for-gestational-age newborns, most had been exposed to chemotherapy or combined treatment during pregnancy.

Only one case of spontaneous preterm labor was recorded. Cesarean section was the predominant delivery mode, with indications related either to maternal oncologic status or fetal conditions. Neonatal complications included respiratory distress and prematurity, with six newborns requiring NICU admission (Table 4).

### 3.6. Survival Analysis

Survival outcomes were assessed using the Kaplan–Meier method, and differences between groups and cancer types were analyzed using the log-rank test. The estimated overall survival probability at 1, 3, and 5 years after cancer diagnosis was 94.7%, 80.2%, and 70.9%, respectively. Group 2 exhibited significantly better overall survival compared to Group 1, with five-year survival rates of 100% and 56.8%, respectively (*p* = 0.036) (Table 5).

**Figure 1:**
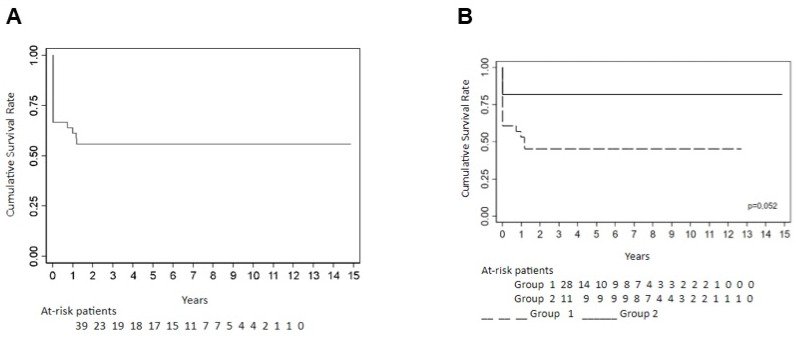
(**A**) Event-free survival function; (**B**) event-free survival function by group.

**Figure 2:**
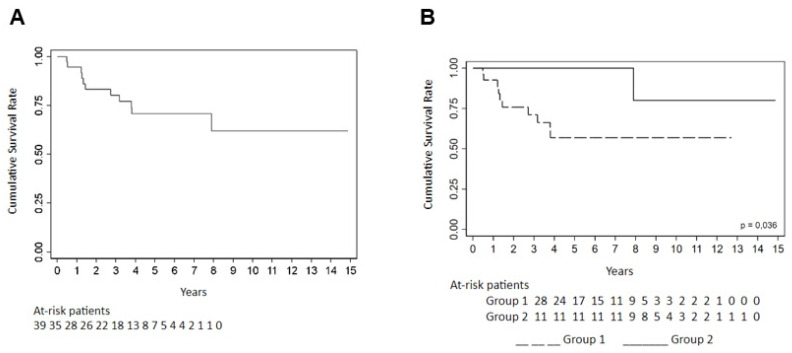
(**A**) Survival function—general death; (**B**) survival function—overall death by group.

The mean overall survival for the entire cohort was 10.5 years (95% CI: 8.4–12.7). Patients in Group 2 had a longer mean survival (13.5 years; 95% CI: 11.0–15.9) than those in Group 1 (8.1 years; 95% CI: 5.9–10.3). No statistically significant differences in overall survival were observed between cancer types (*p* = 0.224), although patients with breast cancer had the lowest five-year survival rate (54.6%), while survival among patients with hematologic, ovarian, and bladder cancers reached 100% (Figure 1 and Figure 2).

Disease-free survival (DFS) was also evaluated. The probability of DFS at 1, 3, and 5 years was 61.3%, 55.9%, and 55.9%, respectively. Although the difference in DFS between groups did not reach statistical significance (*p* = 0.052), a significant difference was observed among cancer types (*p* = 0.006), with breast cancer associated with shorter DFS. The mean DFS for the entire cohort was 8.4 years (95% CI: 6.1–10.7), with a longer mean DFS in Group 2 (12.2 years; 95% CI: 8.8–15.6) compared to Group 1 (5.9 years; 95% CI: 3.6–8.2) (Table 5, Figure 1 and Figure 2).

## 4. Discussion

The increasing global incidence of cancer, particularly among women of reproductive age, has contributed to a growing number of pregnancy-associated cancer cases. While the international literature has addressed this topic, studies focusing on low- and middle-income countries remain scarce. Unlike many international studies that only include cases diagnosed during pregnancy, the present cohort also comprised women who conceived during cancer treatment or follow-up. This broader inclusion reflects real-world clinical scenarios and aligns with the findings of Haan et al. [14], expanding the understanding of maternal and perinatal outcomes in both contexts.

Among women diagnosed during pregnancy (Group 1), more than half did not plan the pregnancy, consistent with the results of Puzzi-Fernandes et al. [15], who reported even higher rates of unplanned pregnancies in women with active cancer. The average maternal age in this cohort was slightly lower than in European studies [14,16], and the population was more racially and socioeconomically diverse. These demographic factors may partially explain the differences in cancer type and stage at diagnosis.

Cervical cancer accounted for 33.4% of cases—significantly higher than in cohorts from high-income countries, where it typically represents less than 10% of pregnancy-associated malignancies [16]. This discrepancy likely reflects disparities in cervical cancer screening and diagnosis, consistent with GLOBOCAN estimates that over 85% of cervical cancer cases occur in low- and middle-income countries [17]. Furthermore, 53.8% of cervical cancer cases in this cohort were diagnosed at advanced stages (II or higher), echoing findings by Vizcaino et al. [18] in resource-limited regions.

Breast cancer was the most frequently diagnosed malignancy, with a high proportion of advanced-stage disease (57.1% in stages III–IV). This rate contrasts with the 30% reported in studies from Europe and North America [16,19]. Possible explanations include physiological changes during pregnancy that obscure early signs, delayed investigation, and limited access to diagnostic services in public health systems. Prior studies, such as García-Manero et al. [20], have shown that these factors can delay diagnosis by up to two months.

The predominance of advanced-stage diagnoses in this cohort likely reflects multifactorial barriers to early detection in Brazil. Sociocultural factors such as low health literacy, fear or stigma associated with a cancer diagnosis, and the prioritization of family responsibilities over self-care may contribute to delayed health-seeking behavior among women of reproductive age. Structural challenges within the healthcare system—including long wait times for specialist referrals, regional disparities in access to diagnostic imaging and pathology, and limitations in screening program coverage—further exacerbate delays in diagnosis. These issues are particularly relevant in the context of pregnancy, where symptoms such as breast changes or vaginal bleeding may be misattributed to gestational physiology, both by patients and healthcare providers. Addressing these barriers requires not only improved access to care but also targeted education and system-level strategies to ensure timely oncologic evaluation in pregnant and reproductive-age women.

In contrast, all ovarian cancer cases were diagnosed at early stages, likely due to incidental detection during routine prenatal ultrasound, as described by Zhao et al. [21]. Early-stage diagnosis enabled conservative surgical approaches in the second trimester that preserved both pregnancy and maternal prognosis [22].

The woman with bladder cancer was diagnosed at an early stage. Due to her advanced age and hematuria, she underwent cystoscopy with biopsy in the 5th week of pregnancy, which confirmed that her cancer was still at an early stage (Ta) [23].

Chronic myeloid leukemia was the most common hematological tumor, but this disease is not classified into stages. As for the two cases of Hodgkin’s lymphoma, staging was available in only one medical record (IVB); however, despite the advanced staging, both women had disease with indolent behavior [24].

One patient in this cohort underwent inadvertent exposure to radiotherapy during early pregnancy, prior to diagnosis confirmation. The treatment occurred between the 7th and 9th gestational weeks, during the organogenesis period, when the fetus is most susceptible to teratogenic effects. Despite the exposure, the newborn was delivered without congenital anomalies. This case reinforces the importance of excluding pregnancy prior to initiating oncologic treatments involving ionizing radiation. Kal and Struikmans [25] emphasize that fetal radiation doses above 100 mGy during the first trimester may lead to spontaneous abortion, growth restriction, malformations, or neurodevelopmental impairment, depending on timing and dosage. While radiotherapy is generally contraindicated during early pregnancy, recent studies suggest that, in selected cases such as breast cancer, it may be performed safely during the first or second trimester using appropriate abdominal shielding to minimize fetal exposure [26]. These findings highlight the need for individualized risk–benefit assessment and careful multidisciplinary coordination when planning cancer treatment in women of reproductive age.

In this cohort, twenty-one women underwent chemotherapy during pregnancy, including three patients who received treatment during the first trimester. Two of these women received adriamycin and cyclophosphamide for breast cancer, and one received interferon-alpha (IFN-α) for chronic myeloid leukemia. Chemotherapy during the first trimester is generally discouraged due to the elevated risk of congenital anomalies, with an estimated incidence of 17–23% depending on the regimen used [27,28]. When treatment is initiated after the first trimester, the teratogenic risk significantly decreases. Anthracyclines, particularly doxorubicin, have demonstrated favorable fetal safety profiles when administered from the second trimester, without evidence of congenital malformations or cardiotoxicity in exposed children, as shown in previous studies [29]. In this study, carboplatin was used in both cervical and ovarian cancer cases. Although rarely used in cervical cancer, carboplatin is frequently administered in pregnancy for epithelial ovarian cancer and is associated with a more favorable toxicity profile than cisplatin, particularly regarding nephrotoxicity, gastrointestinal side effects, and neuropathy [30,31]. These findings support the feasibility of selected chemotherapy regimens during pregnancy, especially when guided by experienced multidisciplinary teams.

Surgical procedures were performed in 41% of patients in this cohort, including both diagnostic and therapeutic interventions. This proportion is consistent with findings from previous studies, such as de Haan et al. [14] and Van Calsteren et al. [16], which reported surgical rates of 39% and 22.1%, respectively, among pregnant women with cancer. In the present study, most procedures occurred during the second trimester (66.7%), a period considered safer for both the mother and fetus due to the reduced risk of miscarriage and preterm labor. Surgical management varied according to tumor type and stage. Among patients diagnosed with breast cancer during pregnancy, 63.6% underwent surgery, including mastectomies, quadrantectomies, and sentinel lymph node biopsies. One patient initially treated with quadrantectomy required subsequent mastectomy and axillary dissection due to disease progression. These results align with those reported by Gomez-Hidalgo et al. [19], in whose study 72% of pregnant women with breast cancer underwent surgical treatment as the first-line approach. The choice and timing of surgical intervention were tailored to gestational age, oncologic status, and maternal condition, highlighting the critical role of multidisciplinary planning in optimizing both maternal and fetal outcomes.

Perinatal outcomes in this cohort were generally favorable. Most deliveries occurred at term (65.8%), and 86.5% of newborns were classified as appropriate for gestational age. The prematurity rate observed (34.2%) was lower than those reported in several international series, such as Van Calsteren et al. [16] (54.2%) and de Haan et al. [14] (48%), and comparable to the findings of Puzzi-Fernandes et al. [15], who reported a term delivery rate of 70.5% in a Brazilian cohort of women with cancer during pregnancy. In the present study, only one case of spontaneous preterm labor was recorded; most preterm births were medically indicated to balance maternal oncologic needs with fetal maturity.

The incidence of small-for-gestational-age (SGA) newborns was 13.5%, a rate similar to that observed by Van Calsteren et al. [16] (14.9%) and lower than that reported by de Haan et al. [14] (21%). In this cohort, most SGA newborns had been exposed to chemotherapy, surgery, or both. Regarding congenital anomalies, the incidence was 7.9%, which is slightly higher than the 4.1% reported by Van Calsteren et al. [16] and the 4% found by de Haan et al. [14] Although this study did not establish a direct causal link between fetal malformations and specific treatments, some of the affected newborns had been exposed to chemotherapy, highlighting the need for continued monitoring and further research in this area.

Six neonates required admission to the neonatal intensive care unit (NICU), primarily for respiratory support due to prematurity. The majority were discharged with their mothers, and only one neonatal death was reported. These findings support the growing body of evidence that cancer treatment during pregnancy—particularly when initiated after the first trimester and coordinated by a multidisciplinary team—can be conducted with acceptable fetal risk. The relatively low rates of prematurity and fetal growth restriction and the limited number of congenital anomalies reinforce the importance of individualized care and careful obstetric–oncologic planning to optimize both maternal and neonatal outcomes.

Survival outcomes in this cohort varied according to cancer type and disease stage. The estimated five-year overall survival was 70.9%, with lower survival observed among patients with breast cancer (54.6%) and more favorable outcomes among those with hematologic, ovarian, and bladder tumors. Although the differences were not statistically significant, the lower survival among breast cancer cases is consistent with prior evidence suggesting poorer prognosis in pregnancy-associated breast cancer. A meta-analysis by Azim et al. [32] demonstrated that women diagnosed with breast cancer during pregnancy have significantly worse overall survival compared to nonpregnant counterparts, even after adjustment for stage and treatment delays. These findings may reflect differences in tumor biology, diagnostic delays, or limitations in therapeutic strategies during gestation.

When interpreted in the context of population-level data, the survival rates observed in this study also echo broader disparities in cancer outcomes. The CONCORD-2 study [33], which assessed global cancer survival across 67 countries, reported five-year survival rates for breast cancer exceeding 85% in high-income countries, while rates in Brazil and other Latin American nations remained below 60%. These contrasts highlight the ongoing impact of structural inequalities in access to early diagnosis and specialized care. In this cohort, the predominance of advanced-stage presentations likely contributed to the lower survival estimates and underscores the importance of investing in public health policies aimed at early detection, continuity of oncologic care, and multidisciplinary support, particularly for women of reproductive age.

This study’s strengths include the long follow-up period, comprehensive clinical and obstetric data, and the inclusion of under-represented populations from Latin America. However, limitations must be acknowledged. Selection bias may have occurred, particularly due to the exclusion of 28 cases with incomplete medical records. These exclusions may have disproportionately involved more complex or severe clinical scenarios, potentially influencing the observed distribution of outcomes. Additionally, missing data limited the ability to perform multivariate analyses or assess certain variables uniformly across all cases. The single-center design may also restrict generalizability, especially in regions of Brazil with different patterns of healthcare access and oncology service availability.

These findings support the feasibility of oncologic treatment during pregnancy in selected cases, particularly when care is coordinated by a multidisciplinary team. The data also underscore the need to improve access to cancer diagnosis and prenatal care in middle-income settings. In particular, strengthening cervical cancer screening during prenatal visits may contribute to earlier detection and improved maternal outcomes.

Future studies should aim to include multicenter cohorts and prospective data collection to improve generalizability and accuracy. Variables such as time to diagnosis, treatment delays, access to multidisciplinary care, and long-term neurodevelopmental outcomes in exposed children warrant particular attention. In middle-income countries, understanding how social determinants of health influence cancer care during pregnancy is also crucial to guide policy and improve equity in maternal–fetal outcomes.

## 5. Conclusions

In this cohort of pregnant women with cancer managed at a single Brazilian referral center, breast and cervical cancers were the most frequent malignancies, often diagnosed at advanced stages. Despite these challenges, maternal and perinatal outcomes were generally favorable, with the majority of deliveries occurring at term and most newborns being appropriate for gestational age. Cancer treatment during pregnancy, when carefully planned and timed, was not associated with adverse neonatal outcomes in most cases. These findings reinforce the importance of multidisciplinary care and support the feasibility of maintaining pregnancy in selected oncologic scenarios.

This study also highlights significant disparities in cancer stage at diagnosis when compared to high-income countries, underscoring the need to improve early detection and screening strategies in middle-income settings. Clinicians should remain vigilant for cancer symptoms during prenatal care, particularly in populations with limited access to health services.

Future prospective multicenter studies are needed to better characterize maternal and neonatal outcomes by cancer type and stage, and to establish evidence-based guidelines tailored to the realities of low- and middle-income countries.

## Figures and Tables

**Table 1:** Sociodemographic characteristics of pregnant women with cancer.

Sociodemographic Characteristics	Group 1 (*N* = 28)		Group 2 (*N* = 11)		Total Cases (*N* = 39)		
	No of Cases (*N*)	%	No of Cases (*N*)	%	No of Cases (*N*)	%	*p* *
Ethnicity							0.333
White	13	46.4%	8	72.7%	21	53.9%	
Mixed	13	46.4%	3	27.3%	16	41%	
Black	2	7.2%	0	0	2	5.1%	
Marital status							1.000
Single	7	25%	2	18.2%	9	23.1%	
Married	21	75%	9	81.8%	30	76.9%	
State of residence							0.545
São Paulo	25	89.3%	11	100%	36	92.3%	
Other	3	10.7%	0	0	3	7.7%	
Education level							0.013
Incomplete primary school	8	28.6%	0	0	8	20.5%	
Complete primary school	2	7.1%	5	45.5%	7	17.9%	
Incomplete high School	15	53.6%	6	54.5%	21	53.9%	
Complete high school	3	10.7%	0	0	3	7.7%	

* Fisher’s exact test.

**Table 2:** Number of pregnant women with cancer.

Type of Cancer	Group 1 (*N* = 28)		Group 2 (*N* = 11)		Total (*N* = 39)		
	No of Case (*N*)	%	No of Case (*N*)	%	No of Case (*N*)	%	*p* *
Cases (*N*)							0.353
Breast Cancer	11	39.3%	3	27.3%	14	35.9%	
Cervical Cancer	10	35.8%	3	27.3%	13	33.4%	
Ovarian Cancer	2	7.1%	1	9.1%	3	7.7%	
Hematologic Cancer	2	7.1%	4	36.3%	6	15.4%	
Gastrointestinal Cancer	2	7.1%	0	0%	2	5.1%	
Bladder Cancer	1	3.6%	0	0%	1	2.5%	

* Fisher’s exact test.

**Table 3:** Staging of pregnant women with cancer.

Staging vs. Type of Neoplasm	Group 1 (*N* = 28)		Group 2 (*N* = 11)		Total (*N* = 39)		
	No of Cases (*N*)	%	No of Cases (*N*)	%	No of Cases (*N*)	%	*p* *
**Breast cancer**	***N* = 11**		***N* = 3**		***N* = 14**		0.835
Stage I	1	9.1%	1	33.3%	2	14.3%	
Stage II	3	27.3%	1	33.3%	4	28.6%	
Stage III	5	45.4%	1	33.4%	6	42.8%	
Stage IV	2	18.2%	0	0%	2	14.3%	
**Cervical cancer**	***N* = 10**		***N* = 3**		***N* = 13**		0.255
Stage I	3	30%	3	100%	6	46.2%	
Stage II	4	40%	0	0%	4	30.7%	
Stage III	3	30%	0	0%	3	23.1%	
**Ovarian cancer**	***N* = 2**		***N* = 1**		***N* = 3**		1.000
IA Stage	1	50%	1	100%	2	66.7%	
IC3 Stage	1	50%	0	0%	1	33.3%	
**Hematologic cancer**	***N* = 2**		***N* = 4**		***N* = 6**		**-**
BVI	0	0%	1	25%	1	16.7%	
Not applicable	1	50%	3	75%	4	66.6%	
Not available	1	50%	0	0%	1	16.7%	
**Gastrointestinal cancer**	***N* = 2**		***N* = 0**		***N* = 2**		**-**
Stage III	2	100%	0	0%	2	100%	
**Bladder cancer**	***N* = 1**		***N* = 0**		***N* = 1**		**-**
Ta Stage	1	100%	0	0%	1	100%	

* Fisher’s exact test.

**Table 4:** Maternal and perinatal outcomes in pregnant women with cancer.

Patient ID	Cancer Type	Stage	GA at Diagnosis (w)	Treatment	GA at Delivery (w)	Birth Weight	Neonatal Outcome	Maternal Outcome
G1-1	Breast	T1cN0M0	10	Surgery + Chemo	37	SGA	Discharged	Alive
G1-2	Breast	T2N0M0	1	Neoadjuvant Chemo	38	AGA	Discharged	Alive
G1-3	Breast	T3N0M0	17	Surgery	41	AGA	Discharged	Alive
G1-4	Breast	T3N0M0	28	Surgery	39	AGA	Discharged	Death
G1-5	Breast	T3N0M0	2	Surgery + Chemo	35	AGA	Discharged	Alive
G1-6	Breast	T3N1aM0	17	Surgery + Chemo	37	AGA	Discharged	Death
G1-7	Breast	T3N2M0	8	Neoadjuvant Chemo + Surgery	39	SGA	Discharged	Death
G1-8	Breast	T3N3cM0	19	Neoadjuvant Chemo	36	AGA	Discharged	Death
G1-9	Breast	T4bN1M0	23	Neoadjuvant Chemo	35	SGA	NICU	Death
G1-10	Breast	T4bN3aM1	7	Surgery + Chemo	35	SGA	Discharged	Alive
G1-11	Breast	TxNxM1	27	Palliative Chemo	30	AGA	NICU	Death
G1-12	Cervical	IB1	7	Surgery	39	AGA	Discharged	Alive
G1-13	Cervical	IB3	21	None	24	AGA	Death	Death
G1-14	Cervical	IB3	26	Neoadjuvant Chemo	37	AGA	Discharged	Alive
G1-15	Cervical	IIA1	25	Neoadjuvant Chemo	36	AGA	Discharged	Alive
G1-16	Cervical	IIA2	20	Neoadjuvant Chemo	38	AGA	Discharged	Alive
G1-17	Cervical	IIB	13	Neoadjuvant Chemo	34	AGA	Discharged	Alive
G1-18	Cervical	IIB	1	Neoadjuvant Chemo	33	AGA	NICU	Alive
G1-19	Cervical	IIIC1	25	Neoadjuvant Chemo	37	AGA	Discharged	Death
G1-20	Cervical	IIIC1	22	Neoadjuvant Chemo	29	Fetal death	-	Alive
G1-21	Cervical	IIIC1	Postpartum	-	37	AGA	Discharged	Death
G1-22	Ovarian	IC3	13	Surgery + Chemo	37	AGA	Discharged	Alive
G1-23	Ovarian	IA	18	Surgery	41	AGA	Discharged	Alive
G1-24	Leukemia	-	9	Chemo	Abortion	-	-	Alive
G1-25	Lymphoma	-	7	None	37	AGA	NICU	Alive
G1-26	Gastrointestinal	IIIB	27	None	36	AGA	Discharged	Alive
G1-27	Gastrointestinal	T4N0M0	27	None	30	AGA	NICU	Death
G1-28	Bladder	TaG1	5	Surgery	41	AGA	Discharged	Alive
G2-1	Breast	T1bN0M0	Before pregnancy	None during pregnancy	40	AGA	Discharged	Alive
G2-2	Breast	T1N3M0	Before pregnancy	Incidental Radiotherapy	29	SGA	NICU	Death
G2-3	Breast	T2N1M0	Before pregnancy	Surgery + Chemo	37	AGA	NICU	Death
G2-4	Cervical	IB1	Before pregnancy	None during pregnancy	38	AGA	Discharged	Alive

Patient G1-7 discovered the cancer during her first pregnancy and underwent her first neoadjuvant CT; she was then lost to follow-up and became pregnant again when she underwent a mastectomy with axillary dissection. AGA: adequate for gestational age; GA: gestational age; Chemo: chemotherapy; NA; not available; SGA: small for gestational age; RT: radiotherapy; W: weeks; NICU: neonatal intensive care unit.

**Table 5:** Kaplan–Meier survival analysis results for metastasis or death.

Cumulative % Survival							*p* *
	6 Months	1 Year	2 Years	3 Years	4 Years	5 Years	
**Total**	66.67 ± 7.55	61.33 ± 7.83	55.87 ± 8.03	55.87 ± 8.03	55.87 ± 8.03	55.87 ± 8.03	
**Group**							0.052
1	60.71 ± 9.23	53.13 ± 9.51	45.22 ± 9.60	45.22 ± 9.60	45.22 ± 9.60	45.22 ± 9.60	
2	81.82 ± 11.63	81.82 ± 11.63	81.82 ± 11.63	81.82 ± 11.63	81.82 ± 11.63	81.82 ±11.63	
**Type of tumor**							0.006
Gastrointestinal tract	(1)	(1)	(1)	(1)	(1)	(1)	
Bladder	100.00 (-)	100.00 (-)	100.00 (-)	100.00 (-)	100.00 (-)	100.00 (-)	
Cervix	76.92 ± 11.69	76.92 ± 11.69	58.61 ± 14.45	58.61 ± 14.45	58.61 ± 14.45	58.61 ± 14.45	
Breast	42.86 ± 13.23	28.57 ± 12.07	28.57 ± 12.07	28.57 ± 12.07	28.57 ± 12.07	28.57 ± 12.07	
Ovary	100.00 (-)	100.00 (-)	100.00 (-)	100.00 (-)	100.00 (-)	100.00 (-)	
Hematological	100.00 (-)	100.00 (-)	100.00 (-)	100.00 (-)	100.00 (-)	100.00 (-)	

* Log rank test. (1) Absence of cases.

## Data Availability

Data are available in the article and Supplementary Materials.

## Supplementary Materials

The following supporting information can be downloaded at: https://www.mdpi.com/article/10.3390/diagnostics15081012/s1, Table S1. Clinical characteristics and habits of pregnant women with cancer; Table S2. Mean event-free time (years) estimated via Kaplan-Meier model; Table S3. Results of the Kaplan-Meier survival analysis for overall death; Table S4. Mean time (years) to overall survival estimated using the Kaplan-Meier model.
