# Supplementary material for: Maternal and Perinatal Outcomes in Pregnant Women with Cancer: A Single-Center Retrospective Cohort Study
Source: Diagnostics (Basel). 2025 Apr 16;15(8):1012. doi: 10.3390/diagnostics15081012 (PMC12026452; doi:10.3390/diagnostics15081012)
Supplement: Supplementary file 1 [file diagnostics-15-01012-s001.zip › diagnostics-3590837-supplementary.pdf]

Table S1. Clinical characteristics and habits of pregnant women with cancer.

**Clinical characteristics**	**Group 1 (N = 28)**		**Group 2 (N = 11)**		**Total (N = 39)**		
**Body mass index (BMI)**	**Nº of cases (N)**	**%**	**Nº of cases (N)**	**%**	**Nº of cases (N)**	**%**	**p ***
**Breast cancer**	**N = 11**		**N = 3**		**N = 14**		1.000
Obesity	4	36.4%	2	66.7%	6	42,8%	
Overweight	2	18.2%	0	0%	2	14,4%	
Appropriate	5	45.4%	1	33.3%	6	42,8%	
**Cervical cancer**	**N = 10**		**N = 3**		**N = 13**		1.000
Obesity	4	40%	2	66.7%	6	46.1%	
Overweight	2	20%	1	33.3%	3	23,1%	
Appropriate	2	20%	0	0%	3	23.1%	
Low weight	1	10%	0	0%	1	7.7%	
**Ovarian cancer**	**N = 2**		**N = 1**		**N = 3**		1.000
Obesity	1	50%	0	0%	1	33.3%	
Appropriate	0	0%	1	100%	1	33.3%	
Low weight	1	50%	0	0%	1	33.3%	
**Hematologic cancer**	**N = 2**		**N = 4**		**N = 6**		1.000
Obesity	1	50%	0	0%	1	16.6%	
Overweight	0	0%	1	25%	1	16.6%	
Appropriate	1	50%	2	50%	3	50%	
Low weight	0	0%	1	25%	1	16.7%	
**Gastrointestinal cancer**	**N = 2**		**N = 0**		**N = 2**		-
Overweight	1	50%	0	0%	1	50%	
Appropriate	1	50%	0	0%	1	50%	
**Bladder cancer**	**N = 1**		**N = 0**		**N = 1**		-
Appropriate	1	100%	0	0%	1	100%	
**Family history of cancer**							
**Breast cancer**	**N = 11**		**N = 3**		**N = 14**		1.000
Yes	3	27.3%	0	0%	3	21.4%	
No	8	72.7%	3	100%	11	78.6%	
**Cervical cancer**	**N = 10**		**N = 3**		**N = 13**		-

No	10	100%	3	100%	13	100%	
**Ovarian cancer**	**N = 2**		**N = 1**		**N = 3**		0.333
Yes	0	0%	1	100%	1	33.3%	
No	2	100%	0	0%	2	66.7%	
**Hematologic cancer**	**N = 2**		**N = 4**		**N = 6**		-
No	2	100%	4	100%	6	100%	
**Gastrointestinal cancer**	**N = 2**		**N = 0**		**N = 2**		-
Yes	1	50%	0	0%	1	50%	
No	1	50%	0	0%	1	50%	
**Bladder cancer**	**N = 1**		**N = 0**		**N = 1**		-
No	1	100%	0	0%	1	100%	
**Smoking**							
**Breast cancer**	**N = 11**		**N = 3**		**N = 14**		1.000
Yes	3	27.3%	1	33.3%	4	28.6%	
No	7	63.6%	2	66.7%	9	64.3%	
Ex-smoking	1	9.1%	0	0%	1	7.1%	
**Cervical cancer**	**N = 10**		**N = 3**		**N = 13**		0.706
Yes	1	10%	1	33.3%	2	15.3%	
No	6	60%	1	33.3%	7	53.9%	
Ex-smoking	3	30%	1	33.4%	4	30.8%	
**Ovarian cancer**	**N = 2**		**N = 1**		**N = 3**		0.333
Yes	0	0%	1	100%	1	33.3%	
No	2	100%	0	0%	2	66.7%	
**Hematologic cancer**	**N = 2**		**N = 4**		**N = 6**		1.000
Yes	0	0%	1	25%	1	16.7%	
No	2	100%	3	75%	5	83.3%	
**Gastrointestinal cancer**	**N = 2**		**N = 0**		**N = 2**		-
No	2	100%	0	0%	2	100%	
**Bladder cancer**	**N = 1**		**N = 0**		**N = 1**		-
No	1	100%	0	0%	1	100%	

* Exact Fisher test

Table S2. Mean event-free time (years) estimated via Kaplan-Meier model.

**Estimate (95%CI)**	
**Total**	8.4 (6.1 – 10.7)
**Group**	
1	5.9 (3.6 – 8.2)
2	12.2 (8.8 – 15.6)
**Type of tumor**	
Gastrointestinal tract	0.0 (0.0 – 0.0)
Bladder	(1)
Cervix	4.9 (2.8 – 7.0)
Breast	3.8 (0.8 – 6.7)
Ovary	(1)
Hematological	(1)

Table S3. Results of the Kaplan-Meier survival analysis for overall death.

	**Cumulative % survival**						**p** *
	**6 months**	**1 year**	**2 years**	**3 years**	**4 years**	**5 years**	
**Total**	97.37 ± 2.6	94.74 ± 3.62	83.25 ± 6.25	80.17 ± 6.74	70.92 ± 7.80	70.92 ± 7.80	
**Group**							0.036
1	96.3 ± 3.63	92.59 ± 5.04	75.76 ± 8.66	71.02 ± 9.32	56.82 ± 10.46	56.82 ± 10.46	
2	100.00 (-)	100.00 (-)	100.00 (-)	100.00 (-)	100.00 (-)	100.00 (-)	
**Type of tumor**							0.224
Gastrointestinal tract	100.00 (-)	100.00 (-)	50.00 ± 35.36	50.00 ± 35.36	50.00 ± 35.36	50.00 ± 35.36	
Bladder	100.00 (-)	100.00 (-)	100.00 (-)	100.00 (-)	100.00 (-)	100.00 (-)	
Cervix	100.00 (-)	100.00 (-)	70.00 ± 14.49	70.00 ± 14.49	70.00 ± 14.49	70.00 ± 14.49	
Breast	92.86 ± 6.88	85.71 ± 9.35	85.71 ± 9.35	77.92 ± 11.29	54.55 ± 13.78	54.55 ± 13.78	
Ovary	100.00 (-)	100.00 (-)	100.00 (-)	100.00 (-)	100.00 (-)	100.00 (-)	
Hematological	100.00 (-)	100.00 (-)	100.00 (-)	100.00 (-)	100.00 (-)	100.00 (-)	

* Log Rank test

Table S4. Mean time (years) to overall survival estimated using the Kaplan-Meier model.

**Estimate (95%CI)**	
**Total**	10.5 (8.4 – 12.7)
**Group**	
1	8.1 (5.9 – 10.3)
2	13.5 (11 – 15.9)
**Type of tumor**	
Gastrointestinal tract	3.8 (0.3 – 7.3)
Bladder	(1)
Cervix	6.0 (4.1 – 7.9)
Breast	7.2 (4.4 – 9.9)
Ovary	(1)
Hematological	(1)
